# General practice cooperatives: long waiting times for home visits due to long distances?

**DOI:** 10.1186/1472-6963-7-19

**Published:** 2007-02-12

**Authors:** Paul Giesen, Nieke van Lin, Henk Mokkink, Wil van den Bosch, Richard Grol

**Affiliations:** 1 Centre for Quality of Care Research (WOK), Radboud University Nijmegen Medical Centre, KWAZO 114, P.O. Box 9101, 6500 HB Nijmegen, The Netherlands; 2 Department of postgraduate training of general practitioners, Radboud University Nijmegen Medical Centre, VOHA 166, P.O. Box 9101, 6500 HB Nijmegen, The Netherlands; 3 Department of family medicine and general practice, Radboud University Nijmegen Medical Centre, HAG 117, P.O. Box 9101, 6500 HB Nijmegen, The Netherlands

## Abstract

**Background:**

The introduction of large-scale out-of-hours GP cooperatives has led to questions about increased distances between the GP cooperatives and the homes of patients and the increasing waiting times for home visits in urgent cases. We studied the relationship between the patient's waiting time for a home visit and the distance to the GP cooperative. Further, we investigated if other factors (traffic intensity, home visit intensity, time of day, and degree of urgency) influenced waiting times.

**Methods:**

Cross-sectional study at four GP cooperatives. We used variance analysis to calculate waiting times for various categories of traffic intensity, home visit intensity, time of day, and degree of urgency. We used multiple logistic regression analysis to calculate to what degree these factors affected the ability to meet targets in urgent cases.

**Results:**

The average waiting time for 5827 consultations was 30.5 min. Traffic intensity, home visit intensity, time of day and urgency of the complaint all seemed to affect waiting times significantly. A total of 88.7% of all patients were seen within 1 hour. In the case of life-threatening complaints (U1), 68.8% of the patients were seen within 15 min, and 95.6% of those with acute complaints (U2) were seen within 1 hour. For patients with life-threatening complaints (U1) the percentage of visits that met the time target of 15 minuts decreased from 86.5% (less than 2.5 km) to 16.7% (equals or more than 20 km).

**Discussion and conclusion:**

Although home visits waiting times increase with increasing distance from the GP cooperative, it appears that traffic intensity, home visit intensity, and urgency also influence waiting times. For patients with life-threatening complaints waiting times increase sharply with the distance.

## Background

The organisation of out-of-hours primary medical care is changing in many countries. We see more and more large-scale general practice (GP) cooperatives with central triage and sometimes a combination of primary care and accident and emergency (A&E) departments in hospitals [1-6]. These changes are due in part to increased workload and the changing needs and attitudes of general practitioners related to their work [1,5].

Around 2000, primary medical care in the Netherlands was also changing from small groups of practitioners taking turns to be on call out of hours to large-scale GP cooperatives [5-7] (Table 1).

**Table 1:** Features of general practice cooperatives in the Netherlands

• Usually situated near a hospital
• Access via a single regional telephone number
• Access daily from 5 p.m. to 8 a.m. and the whole week-end
• Large-scale handling of 100,000 to 500,000 patients within distances of 20–30 km
• Chauffeurs in recognisable GP cars that are fully equipped (e.g., O_2_, infusion drip, automatic defibrillation equipment).
• ICT support including electronic patient files, electronic feedback to the GPs and on-line connection to the GP car
• Triage nurses in contact by telephone (i.e., GP or hospital nurses)
• General practitioner shifts of 6 to 8 hours

With the introduction of out-of-hours GP cooperatives in the Netherlands, the physical distance between the patient and the general practitioner (GP) increased, especially in rural areas. The question of whether the GP can reach patients in time for very urgent problems has led to social unrest, especially on places at big distances from the GP cooperatives[7]. In 2004, this unrest resulted in an investigation by the Dutch Inspectorate of Health Care (IGZ), which criticized the distribution of out-of-hours GP cooperatives throughout the Netherlands and the large distances between GP cooperatives and patients. The IGZ advocated the setting up of satellite cooperatives[7].

Underlying the social unrest and the IGZ recommendations is the general assumption of a more or less linear relationship between the patients' distance to the GP cooperatives and the patient's waiting times for a home visit in urgent cases[7]. It is not known whether this assumption is correct or if there are other factors that influence waiting times as well. Our review of the Dutch literature and a Medline search did not provide a single article in which the relationship between the distance to the services and the patient's waiting times was studied. A better understanding of this relationship is relevant because it can help us set up guidelines with respect to the organisation of the services, the size of the area for a cooperative, the location of the PG cooperative, the number of available GP cars, and coordination with the ambulance service[8].

Although we also assume that there is a relationship between distance and waiting times, we also hypothesize that other factors, such as traffic intensity, home visit intensity, and time of day, are important in explaining waiting times for home visits. We also expect that the urgency estimated at the telephone may influence waiting times. Further analysis of waiting times of patients with very urgent problems is important because too long waiting times for these patients can lead to permanent damage or even to death.

Therefore we conducted a study aimed at answering the following questions:

• To what extent is waiting time related to patients' distance to the GP cooperative, traffic intensity, home visit intensity, time of day, and the urgency estimated by telephone triage?

• What is the proportion of very urgent consultations (U1 and U2) for which the national time limits are satisfied and to what extent is this related to distance, traffic intensity and home visit intensity?

## Methods

We conducted a cross-sectional study of patient's waiting times for all home visits at four out-of-hours GP cooperatives in the Netherlands in the period 2002–2005. At the four GP cooperatives, there were complaints from the population about the long distances at the time of this study. We did not exclude any of the home visits, and in the case of missing information or none at all, a missing-value code was used. Table 2 shows the characteristics of the participating GP cooperatives.

**Table 2:** Characteristics of the participating GP cooperatives

GP cooperative	A	B	C	D
City population	140,000	23,800	46,000	77,825
Rural population	35,000	79,500	39,350	100,652
Location of the GP cooperative in the area	Central	Peripheral	Peripheral	Peripheral
Greatest distance (km) to the GP cooperative	19	29**	25	28
*Number of GP cars*				
Evening	2	1	1	1*
Night	1	1	1	1*
Daytime in the weekend	2	2**	1	2
Traffic measures	-use of bus lane	Flashing lights Siren Swing-down posts Short cuts Notice of new traffic obstacles	Flashing lights Siren Swing-down posts Short cuts Notice of new traffic obstacles	Flashing lights Siren Swing-down posts for access within city
Emergency number	Yes	Yes	Yes	Yes
Telephone doctor present	Yes	No	No	Yes
Urgency determined by	Triagist + Telephone doctor	Triagist	Triagist	Triagist + Telephone doctor

*During evenings and nights, one GP car is on immediate call from a private address

** During the day on the weekend, the GP car is parked on the perimeter, so that the greatest distance is reduced to 19.6 km

### Procedures

With or without consulting the supervising telephone doctor[9], the triage assistants routinely determined the urgency on the telephone on the basis of the complaint. At post A, the urgency was determined later, after the reading of the complaint, according to a procedure described elsewhere[10]. The time at which the telephone conversation ended and the time of day was registered electronically or by hand (post D). The arrival time was taken from the time registration that was routinely updated by the chauffeurs of the GP cars. For each home visit, the shortest distance between the GP cooperative and patients' address was calculated with the aid of the route planner of the Dutch Automobile Association, the ANWB. We obtained an overview of the intensity of traffic from the traffic police; the overview indicates whether there was off-peak, intermediate or rush hour traffic for every half hour on all days of the week. All consultations were classified into these three categories on the basis of the time when the telephone conversation ended.

### Variables

The *waiting time *for the arrival of the consultation doctor was the dependent variable. This was defined as the time from the end of the telephone conversation to the arrival of the GP car. Table 3 shows the national target values by urgency category [11].

**Table 3:** Urgency classes of the Dutch College of General Practitioners Telephone Guide

*Life-threatening (U1)*. Complaints in which the vital functions are in danger. The assistant informs the GP immediately. The GP interrupts his/her work at once and goes to the patient as quickly as possible; this must be within **15 min**. If necessary, the ambulance service is notified at the same time (e.g. for a complaint with a serious chance of heart attack or loss of consciousness). *Acute (U2)*. Complaints for which there is a real chance that the condition of the patient will worsen in a short time, with a risk of loss of vital functions. The assistant informs the GP immediately. The GP sees the patient as soon as possible, certainly within **1 hour **(e.g. for the rapidly increasing shortness of breath of a patient known to have chronic obstructive pulmonary disease). *Urgent (U3)*. Time plays a potentially negative role for medical or emotional reasons. The patient's condition is evaluated within **3 hours **(e.g. a patient with a cut or a lot of pain). *Routine (U4)*. There is no pressure of time for this request for help. The assistant makes an appointment with the GP or gives information and advice.

The independent variables were:

- *Distance*: the number of kilometres between the GP cooperative and the consultation address. These data were classified in distance categories (0.0–2.4, 2.5–4.9, 5.0–7.4, 7.5–9.9, 10.0–14.9, 15.0–19.9, and ≥ 20.0 km).

- *Traffic intensity*: classified as off-peak, intermediate, or rush hour traffic.

- *Home visit intensity*: the sum of the number of home visits requests in 1 hour before and after each consultation. This was classified as: no visit, one or two visits, or three or more visits.

- *Urgency*: degree of urgency of the complaint as estimated by telephone triage. The urgency was divided into four classes according to the urgency system of the Dutch College of General Practitioners (NHG) Telephone Guide (Table 3) [11].

- *Time of day*: the moment at which the patient approached the GP cooperative, which was, according to a dossier check, in the evening (5 p.m. – 11 p.m.), at night (11 p.m. – 8 a.m.), or during the day on the weekend (8 a.m. – 5 p.m.).

### Analysis

In order to answer the first question, we calculated waiting times by means of a variance analysis (F test) in the various categories of distance, intensity of traffic, consultation business and urgency.

To answer the second question, we calculated waiting times in the various urgency categories by means of a variance analysis. The percentages that met the national time limits were also calculated. For the consultations with the greatest urgency (U1 and U2), we determined, by means of a multiple logistic regression-analysis, which factors were associated with meeting, or not meeting, the time limits (U1 within 15 min and U2 within 60 min). For these calculations, *P *< 0.05 was considered significant

## Results

### Relationship of waiting times to distance

For the 5827 home visits included in the study, the average waiting time was 30.5 min. The waiting time increased linearly with respect to the distance. Patients living 20 km or more from the GP cooperative had to wait an average of 13.4 min longer for a home visit than patients living in the immediate neighbourhood of the GP cooperative (Table 4).

**Table 4:** Relationships of average waiting time to distance, traffic intensity, and home visit intensity, time of day and urgency

	Number of consultations	Average waiting time in minutes	Standard deviation	Significance
Total	5827	30.5	27.4	
*Distance in km*				0.00
0.0–2.4	1326	26.6	28.5	
2.5–4.9	1673	28.6	28.2	
5.0–7.4	842	31.7	28.4	
7.5–9.9	610	30.3	25.9	
10.0–14.9	616	33.7	25.4	
15.0–19.9	505	36.6	23.1	
≥ 20.0	255	40.0	23.1	
*Traffic intensity*				0.00
Off-peak hours	2083	28.2	25.6	
Intermediate hours	2487	31.2	27.8	
Rush hours	1270	32.8	28.9	
*Home visit intensity*				0.01
No visit	1336	22.8	17.4	
1 or 2 visits	2836	29.9	26.4	
≥ 3 visits	1600	37.9	33.2	
*Time of day*				0.00
Evening	2685	29.9	25.6	
Night	1495	25.0	21.7	
Daytime in the weekend	1658	36.4	32.9	
*Urgency*				0.00
U1, Life-threatening	205	13.9	11.3	
U2, acute	1613	23.1	18.5	
U3, urgent	1915	33.1	28.7	
U4, routine	1845	36.2	30.9	

### Factors that influence waiting times

The average the home visit time increased from 28.2 min in the off-peak hours to 32.8 min in rush hours. If there were no other home visits, then the average waiting time was 22.8 min, but the average waiting time could be as much as 37.9 min at very busy times. The waiting time was 25.0 min at night, and could be as much as 36.4 min during the day on the weekend. The waiting time was on average 13.9 min for requests for help that were estimated to be very urgent (U1), and if the urgency was estimated as low (U4), then the waiting time was 36.2 min (Table 4).

### Waiting times and time targets

Altogether, 88.7% of all patients were seen within 60 min. For life-threatening complaints (U1), 68.8% of the patients were seen within 15 min, and 95.6% of the patients with acute complaints (U2) were seen within 1 hour. Of the patients with urgent complaints (U3), 98.4 % were seen within 2 hours, and 100% were seen within the 3-hour time limit (Table 5).

**Table 5:** Home visits with waiting times and time targets for the arrival of the home visit doctor

Urgency	Number of Home visits	% visit ≤ 15 min	% Consultation ≤ 30 min (%)	Consultation ≤ 60 min (%)	Consultation ≤ 120 min (%)**
U1	205	68.8*	95.6	98.5	100
U2	1613	41.2	76.6	95.6*	99.6
U3	1915	29.8	61.4	89.8	98.4*
U4	1845	23.6	56.3	84.3	97.3

Total	5578	32.5	65.4	88.7	98.6

U1, Life-threatening; U2, acute; U3, urgent; U4, routine

*Time targets: 15 min for U1, 60 min for U2, 180 min for U3, and no time limit for U4

**Although the time limit for U3 is 180 min, almost 100% of the U3 patients received a consultation within 120 min. For this reason we chose to maintain the time limit of 120 min

For the patients with life-threatening complaints (U1), the time limit of 15 min appeared to be met significantly less often as the distance increased. The percentage of visits that met the time target decreased from 86.5% near the GP cooperative to 16.7% at a distance 20 km or more [odds ratio (OR) decreasing from 29.9 to 1.6]. All other factors (traffic intensity, home visit intensity, and time of day) did not lead to a significant odds ratio for the U1 category.

In the U2 category, the distance appeared to have no significant influence on waiting times, and approximately 95% of the patients were seen within an hour. Furthermore, the time target was met more often in the U2 category as the number of home visits decreased [no home visits: OR 8.9, confidence interval (CI) 3.0–26.2; and 1–2 home visits: OR 2.8, CI 1.7–4.7; (Table 6).

**Table 6:** Multiple logistic regression-analysis. Relationships of meeting the time targets of the urgency categories U1 and U2 to distance, traffic intensity, home visit intensity, and time of day

	Urgency category: life-threatening (U1)			Urgency category: acute (U2)		
	Number of	Percentage of consultations in ≤ 15 min	Odds ratio and 95% confidence interval**	Number of consultations	Percentage of consultations in ≤ 1 hour	Odds ratio and 95% confidence interval**

Total	204	68.8		1613	95.5	
*Distance in kilometres*						
0.0–2.4	52	86.5	29.9 (2.8–314.2)*	427	96.2	1.5 (0.4–5.4)
2.5–4.9	61	80.3	17.7 (1.8–178.8)*	440	95.9	1.6 (0.5–5.9)
5.0–7.4	34	70.6	12.0 (1.1–126.4)*	235	93.6	1.1 (0.3–3.9)
7.5–9.9	20	55.0	5.3 (0.5–57.7)	190	94.7	1.0 (0.3–3.8)
10.0–14.9	12	33.3	2.1 (0.2–26.5)	121	95.9	2.3 (0.4–11.9)
15.0–19.9	19	31.6	1.6 (0.1–19.0)	137	96.4	1.4 (0.3–6.0)
≥ 20.0	6	16.7	Reference	63	95.2	Reference
*Traffic intensity*						
Off-peak hours	83	74.7	2.1 (0.6–5.2)	622	96.3	1.2 (0.6–2.4)
Intermediate hours	77	64.9	1.1 (0.7–2.8)	669	95.5	1.4 (0.8–2.6)
Rush hours	45	64.4	Reference	322	94.1	Reference
*Home visit intensity*						
No consultations	55	72.7	1.8 (0.6–5.2)	386	99.0	8.9 (3.0–26.2)*
1 or 2 consultations	107	71.0	1.7 (0.7–4.0)	830	96.6	2.8 (1.7–4.7)*
≥ 3 consultations	42	57.1	Reference	380	90.3	Reference
*Time of day*						
Evening	86	69.0	1.6 (0.7–3.9)	764	95.5	0.6 (0.2–1.4)
Night	69	74.3	1.4 (0.4–4.9)	495	97.4	1.7 (0.7–4.0)
Daytime in the weekend	47	59.6	Reference	353	92.9	Reference

*P < 0.05

**Interpretation: the greater the odds ratio is, the greater the chance that the patient will be seen within the time limit

## Discussion and conclusion

### Main findings

The average waiting time for all home visits was half an hour, and almost 90% of all home visits took place within an hour. Traffic intensity, home visit business, and urgency of the complaint all had a significant influence on this waiting time. Seventy percent of all patients with an urgency of U1 were seen within 15 min, and 95% of all patients with an urgency of U2 were seen within an hour. For patients with life-threatening complaints (U1) the time target was met increasingly less often as the distance increased. This appeared not to apply for U2, for which waiting times and distance were not related, but for which the home visit business significantly influenced whether the time target was met.

### What this study adds

Patients with a U2 or U3 classification were seen so well within the time target that, as this study indicates, the time target for U2 cases could be reduced to 1/2 hour and the time target for U3 cases could be reduced to 2 hours. The short patient waiting times for home visits can possibly be explained by the fact that the house call GP has no other duties and can therefore carry out the consultations without interruption. The driver possibly makes a contribution to shorter waiting times by being aware of the traffic situation and by taking measures to get there faster, by using the bus lane, for example.

The patient's waiting time is largely determined by the urgency category. Training in correctly classifying the urgency is therefore very important to ensure that the right patient receives the right care at the right moment.

The time target of 15 min for patients with life-threatening complaints (U1) appears to be met significantly less often as the distance increases. Furthermore, it appears that other factors, such as traffic intensity and home visit business, are of hardly any influence. This is probably due to the fact that the doctor interrupts his work immediately for a U1 patient and uses the bus lane, sirens, and flashing lights to get to the patient immediately. For a somewhat lower priority, such as that for U2, we see that distance does not play a role, but home visit business and traffic intensity do.

How, then, can we gain time for patients with life-threatening complaints (U1)? Although literature about this subject is lacking, we can, on the basis of this study, cautiously suggest that the distance to the patient be shortened by spreading the starting points of the GP cars and ambulances over the work area in as well balanced a way as possible. Further, it is very important that the GP cooperatives and ambulance services complement each other as seamlessly as possible by means of agreements about mutual fine tuning of times and efforts [7-9].

### Limitations

We do not know of any published study about waiting times for consultations, so we cannot compare our data with those of others. However,. the results of the subsets of GP cooperatives proved to be almost similar (split-half method). This strengthens the idea that the results can be generalized to some degree. However, each district has its own unique characteristics that influence waiting times. For example, there is a large suburb 5 km from GP cooperative A, that is difficult to access because of traffic bumps and roundabout routes. This caused a sharp increase in waiting times for the patients, which made it comparable to the waiting times at a distance of 20 km (data not shown).

A limitation of this study is that there were relatively few patients with life-threatening complaints, so that results pertaining to them should be interpreted with caution.

### Implications for research

Further research is indicated regarding models of more cooperation between GP cooperatives and ambulance services with a view to how waiting times for patients with life-threatening complaints can be reduced. Also the question of what the consequences are for the patient if the U1 time limit of 15 min is not met should also be studied.

In this cross-sectional study, we have studied the patient's waiting times to see the home visit GP. Attention for waiting times is important in order to assure that the patient receives the right care at the right moment.

## Competing interests

The author(s) declare that they have no competing interests.

## Authors' contributions

PG and NL designed and carried out the study. HM supervised the study methodologically and performed the analysis. WB and RG supervised the study and revised the manuscript. All authors read and approved the final manuscript.

## Pre-publication history

The pre-publication history for this paper can be accessed here:


